# Ashwagandha as a Unique Cause of Thyrotoxicosis Presenting With Supraventricular Tachycardia

**DOI:** 10.7759/cureus.23494

**Published:** 2022-03-25

**Authors:** Hawra I Kamal, Kunjal Patel, Alexandra Brdak, Jeremy Heffernan, Naseer Ahmad

**Affiliations:** 1 Internal Medicine, Ascension Providence Hospital, Southfield, USA; 2 Internal Medicine, Ascension Providence Hospital, Novi, USA; 3 Endocrinology, Diabetes and Metabolism, Ascension Providence Hospital, Southfield, USA

**Keywords:** herbal supplement, supraventricular tachycardia, hyperthyroidism, thyrotoxicosis, ashwagandha

## Abstract

Ashwagandha root extract (ARE) is a reputed herbal supplement in Ayurvedic medicine for a variety of health conditions. To date, scant attention has been paid to thyrotoxicosis associated with Ashwagandha and it is rarely reported in the literature. We report a 73-year-old female who presented with supraventricular tachycardia, symptoms of hyperthyroidism and significantly low TSH levels, after two years of using ARE as a self-administered treatment for hypothyroidism. Full symptomatic resolution and biochemical improvement ensued upon cessation of the supplement. Though the pathophysiology remains obscure, we hope to promote awareness concerning this rare yet possible side effect of Ashwagandha.

## Introduction

Ashwagandha root extract (Withania somnifera) has been widely used for centuries in Ayurvedic medical practice in India as an antioxidant, anti-cancer, anxiolytic, and antidepressant agent [1]. Despite decades of research on W. somnifera, data on its effect on thyroid function have been less than satisfactory. A growing literature has hinted at a link between ARE use and increase in thyroid hormones, however, the available data remain contradictory. While few, small randomized controlled trials (RCTs) found that ARE effectively normalized the serum TSH and was a safe treatment among patients with hypothyroidism, there remain concerns with thyrotoxicosis as a reported side effect [2,3].

Thyrotoxicosis refers to excess circulating thyroid hormone levels in the body resulting in multitudinous clinical presentations and hence will be encountered by physicians in all medical specialties. The symptoms of thyrotoxicosis may range from palpitations, weight loss, heat intolerance, fine tremors, anxiety to more serious cardiac arrhythmias and heart failure. It is important to determine the etiology of thyrotoxicosis, as this determines management. Untreated thyrotoxicosis can lead to life-threatening complications including cardiac arrhythmias or thyroid storm.

Hereby, we present a case of a patient with hypothyroidism presenting with supraventricular tachycardia ultimately diagnosed with new-onset thyrotoxicosis after two years of ARE use. This article delves into the events that led to the diagnosis and concurrently illustrates a number of unique learning pearls.

## Case presentation

A 73-year-old female with a history of primary hypothyroidism presented to the emergency department with acute onset palpitations, chest pain and shortness of breath. She was found to have convincing clinical symptoms of thyrotoxicosis including tremor, tachycardia, palpitations, dizziness, fatigue, irritability, loose stools, and hair thinning over the last few weeks. There was no past medical history of ischemic heart disease, hypertension or heart failure. The patient did have a history of hypothyroidism for which she was taking levothyroxine and she stopped taking it two years prior. Instead, she started taking ARE as an alternative medicine for hypothyroidism. No history of beta-2 agonists, atropine, or theophylline use was obtained. She denied any history of smoking cigarettes, marijuana, excessive intake of caffeine, alcohol or illicit drug use. She also denied any recent viral infection or exposure to iodinated contrast. Blood pressure was 121/90 and heart rate was 173 beats per minute (BPM). She had no evidence of thyroid enlargement, thyroid nodules, or tenderness of the thyroid gland on physical examination.

**Figure 1:**
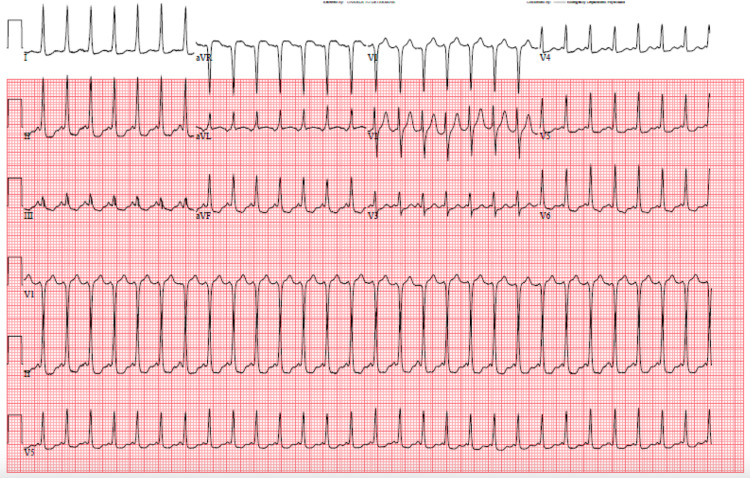
Initial EKG showing SVT with HR 173 BPM

**Figure 2:**
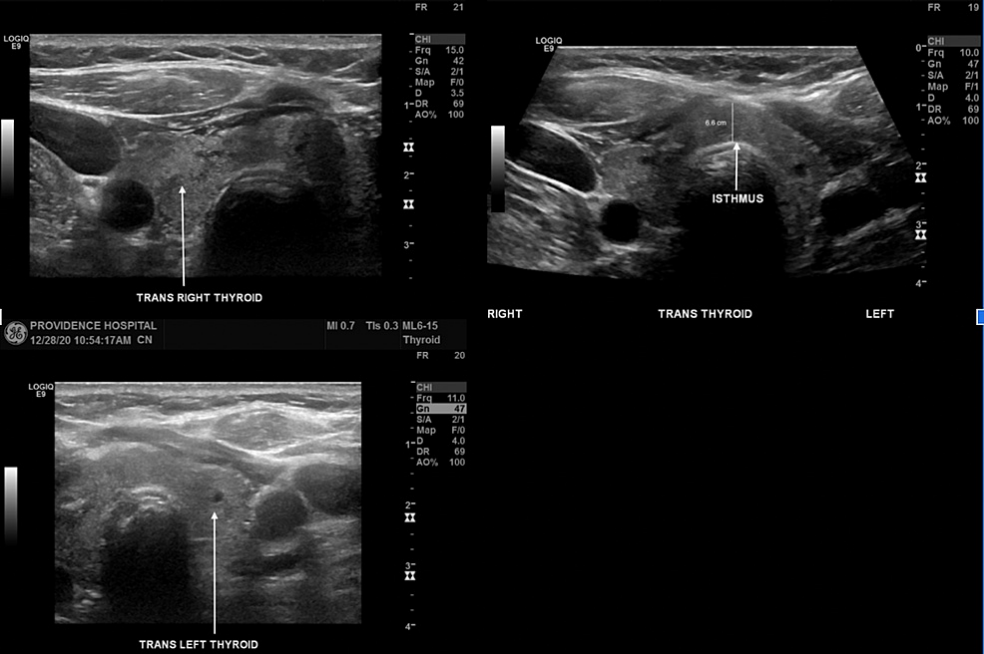
Thyroid ultrasound showing mildly heterogeneous gland with thickened isthmus without a discrete suspicious thyroid nodule

Initial EKG revealed a narrow QRS complex with a heart rate of 173 BPM consistent with supraventricular tachycardia (SVT) (Figure 1). Serial cardiac enzymes were negative on three repeat draws. Laboratory investigations revealed markedly low TSH of <0.01 mcIU/mL (0.27- 4.20 mcIU/mL) and normal free T4 of 0.87 ng/dL (0.80- 1.70 ng/dL) and total T3 of 97.7 ng/dL (​​80.0- 200.0 ng/dL) (Table 1). Additional labs were obtained and showed thyroid-stimulating immunoglobulin and thyroglobulin antibody within normal limits; however, thyroid microsomal antibody was elevated to 471 (Normal <9) representing Hashimoto’s thyroiditis as an etiology of her baseline hypothyroidism. CBC showed normal hemoglobin. Serum ethanol level was not detectable and urine drug screen was negative. Thyroid ultrasound revealed a mildly heterogeneous gland with no discrete nodules (Figure 2). An echocardiogram showed no structural heart disease associated with SVT and it showed normal LV systolic function. A pharmacological (Lexiscan) myocardial perfusion stress test was done after discharge and was negative.

**Table 1:** Thyroid function test results on presentation and on subsequent follow-up visits

Lab indices (Normal range)	On presentation	After 2 weeks	After 5 weeks
TSH (0.27- 4.20 mcIU/mL)	<0.01 L	3.49	4.31 H
Free T4 (0.80- 1.70 ng/dL)	0.87	0.80	0.7 L
Total T3 (​​80.0- 200.0 ng/dL)	97.7	-	-
Free T3 (2.5-4.2 pg/mL)	-	0.70	2.20 L

For acute treatment of the supraventricular tachycardia, ​​adenosine 6 mg, adenosine 12 mg, and diltiazem 10 mg bolus were administered to convert to normal sinus rhythm. She was prescribed oral metoprolol succinate 25 mg daily and was advised to stop taking the Ashwagandha tablets.

**Figure 3:**
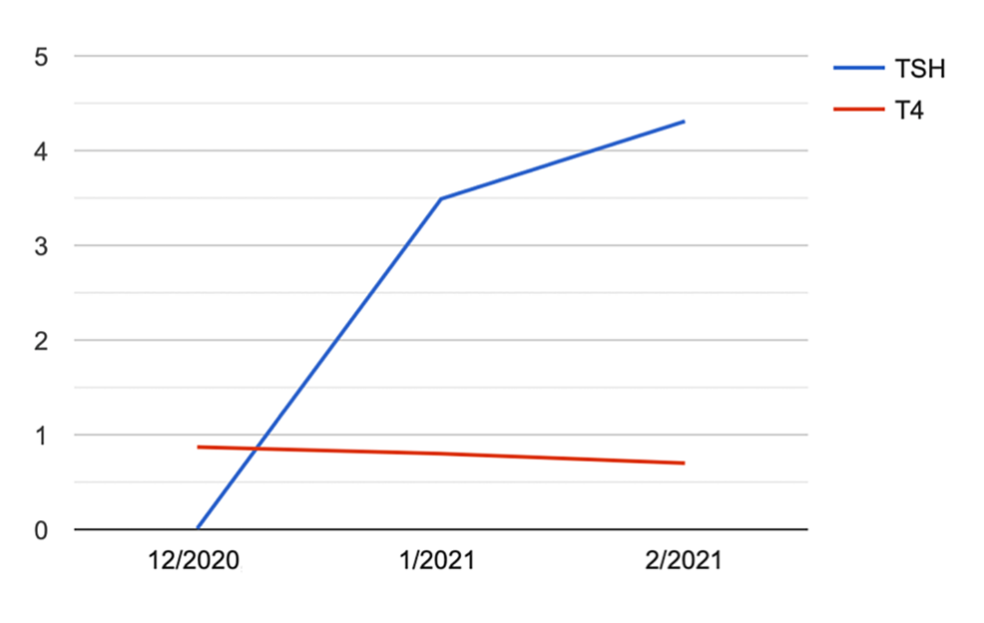
Graph illustrating TSH and T4 trend over a two-month period

At follow-up visit two weeks later, the patient was noted to have a resolution of her symptoms and labs showed TSH within normal limits 3.49 mcIU/mL (0.27- 4.20 mcIU/mL) and free T4 0.8 ng/dL (0.80- 1.70 ng/dL) and free T3 0.7 pg/mL (2.3-4.2 pg/mL) were at the lower limit of normal. Subsequent labs three weeks later showed an elevated TSH 4.31 mcunit/mL (0.27- 4.20 mcIU/mL) and low free T4 of 0.7 (0.80- 1.70 ng/dL) and free T3 of 2.2 pg/mL (2.5-4.2 pg/mL), indicating a return to her baseline hypothyroid status (Figure 3, Table 1). Given the significantly positive thyroid microsomal antibody and ultrasound findings, the etiology of her hypothyroidism seems to be Hashimoto thyroiditis. Her presenting arrhythmia was ultimately attributed to thyrotoxicosis due to Ashwagandha given the temporal relationship between initiation of Ashwagandhas and the onset of symptoms, and resolution upon cessation. Only TSH was suppressed indicating a possible early phase of hyperthyroidism. A possible explanation for the normal free T4 is the underlying Hashimoto thyroiditis which may affect the thyroid tissue's ability to produce thyroid hormones despite the TSH stimulatory effect. When we took her off the ARE, she gradually went to a hypothyroid state again which means the hyperthyroid state was likely from Ashwagandha.

## Discussion

This case suggests that thyrotoxicosis may be associated with Ashwagandha use and it may be considered when there is a temporal relationship between the introduction of Ashwagandhas and the onset of symptoms. Similar to our case, we found two case reports of patients who developed symptomatic thyrotoxicosis supported by laboratory assessment while taking ARE [2,4]. In contrast to our case, patients in other case reports had normal thyroid function before use of ARE and no prior history of hypothyroidism.

Surprisingly, T3 and T4 levels in our patient remained normal, and only TSH was elevated. This is in contrast to other studies that reported a change in T3 and T4 as the main observed effect [2]. A possible reason for this discrepancy might be that our patient had baseline hypothyroidism and she stopped taking her medications two years prior to presentations. Her thyroid function tests before using ARE were unknown to us. Despite that, she presented with symptomatic thyrotoxicosis complicated by cardiac arrhythmias.

One must question the mechanism by which ARE may exert its effect on thyroid status. Although the pathophysiology is not yet entirely known, it is postulated that ARE stimulates thyroidal activity as manifested by an increase in serum T4 and T3 concentrations [5]. More specifically, it is thought that ARE stimulates the synthesis and/or release of T4 at the glandular level. Panda et al. observed that administration of ARE to mice for 20 days resulted in a considerable increase in the level of T4 in serum reaching approximately 111% compared to only an 18% increase in serum T3 [6]. Considering that T4 is the predominant circulating thyroid hormone and is synthesized only in the thyroid gland, researchers proposed that the increase in T4 concentration after administration of ARE suggests that it primarily stimulates the thyroid gland to synthesize and/or secrete thyroxine [5,6]. In addition to that, Kang et al. found evidence that commercially available supplements including Ashwagandha, contain amounts of T3 and T4 that exceed the doses required to treat hypothyroidism, exposing patients to a risk of iatrogenic thyrotoxicosis. They examined 10 thyroid dietary supplements including herbal-based supplements containing Ashwagandha, for the presence of both thyroxine (T4) and triiodothyronine (T3), and found that the majority of dietary thyroid supplements contained clinically relevant amounts of T4 and T3 [7].

Our case raises intriguing questions about whether ARE is safe enough to be used in the treatment of hypothyroidism as claimed by several RCTs and whether we need to monitor thyroid function testing before and during ARE use. For instance, while not conclusive, there is some evidence that ARE can be used safely even in the treatment of subclinical hypothyroidism. Prior RCTs were conducted to examine the safety of ARE by measuring TSH, T3, and T4 at baseline and after eight weeks [3,8]. However further studies including larger samples and longer follow-up times are required.

## Conclusions

While our case illustrates a potential cause of thyrotoxicosis that is reversible and not yet widely well investigated, it also sheds light on the importance of obtaining a detailed medication history including herbal supplements, obtaining a complete review of systems, and always keeping a broad differential diagnosis. This case illustrates that herbal medications continue to impose a diagnostic and therapeutic challenge for clinicians. Further research is needed to investigate the effect of Ashwagandha on thyroid function tests and to establish a safe dose range for patients. Patients should be aware of the possible consequences of Ashwagandha on thyroid function.
